# Controlled Oxidation of Metallic Molybdenum Patterns via Joule Heating for Localized MoS_2_ Growth

**DOI:** 10.3390/nano15020131

**Published:** 2025-01-16

**Authors:** Norah Aldosari, William Poston, Gregory Jensen, Maryam Bizhani, Muhammad Tariq, Eric Stinaff

**Affiliations:** 1 Department of Physics and Astronomy Athens, Ohio University, Athens, OH 45701, USA; na314617@ohio.edu (N.A.); wp826418@ohio.edu (W.P.); gj772812@ohio.edu (G.J.); mb795116@ohio.edu (M.B.); mt967721@ohio.edu (M.T.); 2 Nanoscale and Quantum Phenomena Institute (NQPI), Athens, OH 45701, USA; 3 Department of Physics and Astronomy, College of Science and Humanities, Prince Sattam bin Abdulaziz University, 173, Al-Kharj 16278, Saudi Arabia

**Keywords:** 2D materials, transition metal dichalcogenide, transition metal oxide, joule heating, resistive heating, bottom–up fabrication, sulfurization, characterization

## Abstract

High-quality two-dimensional transition metal dichalcogenides (2D TMDs), such as molybdenum disulfide (MoS_2_), have significant potential for advanced electrical and optoelectronic applications. This study introduces a novel approach to control the localized growth of MoS_2_ through the selective oxidation of bulk molybdenum patterns using Joule heating, followed by sulfurization. By passing an electric current through molybdenum patterns under ambient conditions, localized heating induced the formation of a molybdenum oxide layer, primarily MoO_2_ and MoO_3_, depending on the applied power and heating duration. These oxides act as nucleation sites for the subsequent growth of MoS_2_. The properties of the grown MoS_2_ films were investigated using Raman spectroscopy and photoluminescence measurements, showing promising film quality. This study demonstrates that Joule heating can be an effective method for precise control over TMD growth, offering a scalable approach for producing high-quality 2D materials that have the potential to be integrated into next-generation electrical and optoelectronic technologies.

## 1. Introduction

Recent advances in two-dimensional transition metal dichalcogenide (2D TMD) materials have highlighted their significant potential in future electronic and optoelectronic devices, owing to their unique properties and wide range of applications [1,2,3]. These materials have been successfully integrated into various devices, including field effect transistors, memory devices, photodetectors, and photocatalysts for hydrogen evolution reactors [4,5]. Specifically, monolayer semiconducting TMDs exhibit direct band gaps and remarkable optical properties, making them ideal for applications in light-emitting diodes and photovoltaic technologies. For instance, monolayer MoS_2_ transistors have on/off ratios of 10^8^ and an intrinsic band gap of about 1.8 eV [6].

In addition to these applications in electronic and optoelectronic devices, MoS_2_ has emerged as a promising quantum material due to its potential in quantum computing and information processing. Its unique electronic properties, such as quantum confinement and spin–valley coupling, make it an exciting candidate for next-generation quantum technologies. Recent researchers have fabricated MoS_2_ nanotubes and nanoribbons, which exhibit quantum confinement effects critical for qubit operations [7]. Furthermore, thin films, such as MoS_2_, have been explored in terms of their compatibility with scalable device fabrication techniques, offering opportunities to integrate quantum properties into practical applications, such as photonic and spintronic devices. The ability to modify the dimensionality and electronic structure further enhances its versatility in quantum material research [8].

However, the fabrication of high-quality 2D and large-scale transition metal dichalcogenides (TMDs) remains a considerable challenge, despite their significant properties. Scalable fabrication processes are crucial for realizing the potential of TMDs in advanced electrical and optoelectronic applications [1]. Chemical vapor deposition (CVD) has emerged as a highly promising method for synthesizing mono- to few-layer transition metal dichalcogenides (TMDs). The CVD growth process is based on a vapor phase chemical reaction between a transition metal and chalcogen precursors on the substrate surface, which results in the formation of TMD crystals. Although the CVD method can achieve large-scale TMDs thin films, the quality of the resulting material is highly dependent on the precursor resources and often results in non-uniform TMD films [9,10,11,12,13]. However, recent works demonstrate that covering the substrate totally or partly with transition metals, such as Mo foils, or transition metal oxides, such as MoO_3_, can facilitate the growth of highly crystalline 2D TMDs at precise locations. Subsequently, to fabricate functional devices, TMD materials grown using this method require additional steps such as etching, transfer, lithography, and metal deposition. These steps can introduce variability in material characteristics and overall device performance [14,15,16,17,18]. In previous works, we introduced a straightforward and scalable CVD-based method for fabricating as-grown 2D TMD-based devices by promoting the growth of 2D-TMD materials around lithographically defined bulk transition metal patterns [19,20,21,22]. An oxidation step during the CVD process generates a metal oxide layer directly on the surface of the bulk transition metal, eliminating the requirement for a separate transition metal precursor. By adjusting the CVD growth parameters, the composition and structure of the metal oxide layer can be controlled on the surface of the transition metal. This oxide layer serves as both the source of the transition metal and the nucleation site for TMD material formation. During the growth process, the oxide layer is consumed, and the resulting thin-film TMD material spreads outward across the substrate. This approach enables the synthesis of highly crystalline TMD films with domain sizes reaching tens of micrometers [19,20,21,22]. However, since the entire metallic pattern undergoes oxidation, TMD material tends to grow around the entire metallic patterns, which complicates later stages of device fabrication. To further enhance control over the location and quality of TMD growth, an additional study demonstrated a method for the selective oxidation of bulk Mo metals using continuous-wave laser processing to produce localized mono to few layers and high-quality MoS_2_ films [23]. Laser radiation generates localized heating, resulting in the formation of transition metal oxide on the surface of the bulk material, which serves as a precursor and nucleation site, permitting the localized growth of MoS_2_. Raman investigation showed that exposing bulk Mo metal to laser power approaching 5–10 mW for a few seconds leads to MoO_2_ formation, which promotes the growth of high-quality MoS_2_ [23]. However, despite these advances, challenges remain in achieving precise control over oxidation and growth at the nanoscale. In this context, we propose utilizing Joule heating for the selective oxidation of transition metal wires as a novel approach for localized TMD growth. CVD growth has the benefit of simplicity, yet traditional methods often require extended oxide times. In contrast, a faster approach involves direct Joule heating of patterned molybdenum wires on a Si/SiO**_2_** substrate by applying an electric current under ambient conditions. This method has been successfully utilized to synthesize various metal oxide nanostructures, including MoO_3_, CuO, ZnO, Fe_2_O_3_, and V_2_O_5_ nanowires, within just a few seconds to minutes [24].

Joule heating, also known as resistive or ohmic heating, occurs when an electric current passes through a conductive substance and generates heat due to the material’s resistance. This process is regulated by Joule’s law, which states that the heat produced is proportional to the square of the current, the material’s resistance, and the duration of current flow [25,26]. Building on this concept, our study investigates the use of Joule heating to selectively oxidize patterned Mo metallic wires, which subsequently serve as nucleation sites for the lateral growth of 2D MoS_2_ from the metal source toward the substrate. This approach provides precise control over the oxidation process, providing a possible route to incorporate into existing semiconductor fabrication workflows that is scalable for industrial applications. By utilizing Joule heating, we can selectively oxidize specific regions of the patterned metallic wires, simplifying the fabrication process and promoting lateral growth. This technique shows promise for the scalable fabrication of high-quality 2D TMD-based devices, offering control over the synthesis of advanced materials. Furthermore, applying Joule heating to areas on metal contacts enables precise control over the substrate’s thermal budget. This precise thermal management prevents potential damage or changes to substrate properties, enhancing the efficacy of Joule heating in semiconductor fabrication. This work emphasizes the importance of controlling the lateral growth process to achieve high-quality 2D TMD-based devices with advanced material properties and spatial precision.

## 2. Materials and Methods

**Figure 1:**
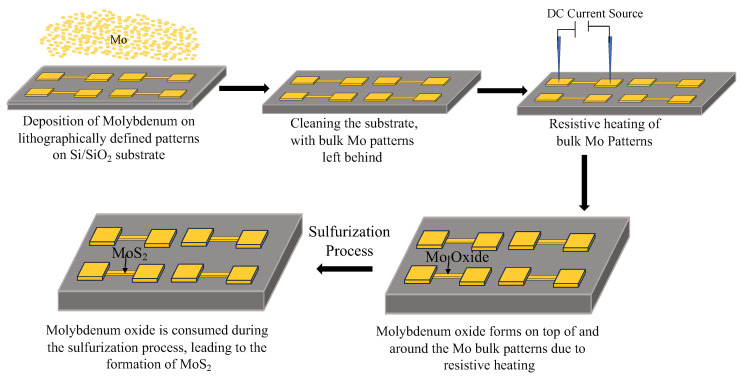
Schematic of the experimental process for the oxidation and growth of MoS_2_. Initially, Mo is deposited onto lithographically defined patterns on a Si/SiO_2_ substrate. Through Joule heating using a DC current source, localized oxidation occurs, forming Mo oxide (MoOx) on top of and around the Mo metallic patterns. During the sulfurization process, the oxide is consumed, leading to the formation of MoS_2_ in the desired regions.

Defined channel patterns, approximately 25 µm wide and 150 µm long, were patterned on a Si/SiO_2_ substrate using a photolithography process. Following development, a bulk molybdenum layer (~450 nm thick) was deposited onto the patterned substrate via DC sputtering (Denton Vacuum DV-502A) under high vacuum conditions. The lift-off process was performed by immersing the substrate in acetone for 15 min at room temperature. For Joule heating treatments, conducted in ambient air, a DC electric generator connected to an Electroglas (EG 1034X) Automatic Wafer Prober was used to pass current through the Mo wires. The applied power ranged from 2 to 4 W, with current durations varying from 2 to 60 s depending on the experimental conditions. The schematic of the setup is illustrated in Figure 1.

Raman spectroscopy was employed to characterize the samples at each stage of the oxidation process, providing detailed insight into the specific oxides present. The Raman spectra of oxidized regions were obtained using a laser source with a wavelength of 532 nm, a power of 1.5 mW, and an integration time of 15 s. The temperature of the wires during the Joule heating was investigated using photoluminescent materials through the method of Photoluminescence Thermometry, a reliable method for temperature detection [27,28]. For this purpose, Er_2_O_3_ powder was specifically utilized in a separate experiment designed to estimate the temperature of the wire. This experiment was conducted independently to avoid interference with the main results, and the wire dimensions were kept consistent with those used in the primary study to ensure comparable conditions. Details of this experiment and the photoluminescence spectra of Er_2_O_3_ powder are provided in the Supplementary Materials.

Following Joule heating, the samples were subjected to a sulfurization process to localize MoS_2_ formation around the oxidized areas. The samples were placed inside a quartz tube with sulfur powder. To minimize oxygen levels and prevent further oxidation during sulfurization, ultra-high-purity argon gas was introduced at a flow rate of 0.5 LPM for 2 h, which was then reduced to 0.1 LPM. Subsequently, the sample was rapidly heated to 750 °C, and vaporized sulfur was introduced for 5 min to facilitate the formation of MoS_2_ on the substrate. The sample was then allowed to cool naturally. Optical images of the grown regions were taken, and the materials were characterized using Raman and photoluminescence spectroscopy.

## 3. Results and Discussion

The manipulation of materials through Joule heating is a well-established and continuously evolving field of study. In the case of transition metal oxides, Joule (resistive) heating has been explored to generate or alter various oxide phases nanomaterials, highlighting its efficiency [24,25].

**Figure 2:**
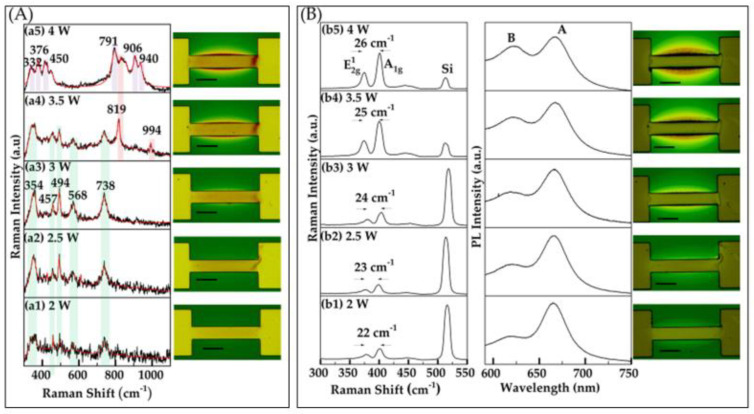
(**A**) Raman characterization and optical images of oxide layers formed on Mo patterns induced by Joule heating. Oxide formation was investigated as a function of applied power, with a fixed duration of 2 s. Raman spectra were recorded with a power of 1.5 mW and an integration time of 15 s. This section includes five sub-panels (**a1**–**a5**) displaying Raman spectra corresponding to different oxide formations from varying applied power levels, along with optical images of the oxide regions. (**B**) Raman, PL spectra, and optical images of the sample after sulfurization. This section includes five sub-panels (**b1**–**b5**), each displaying Raman and PL spectra along with corresponding optical images that illustrate the structural and spectral evolution of MoS_2_. Each optical image features a scale bar representing 50 μm.

In our investigation, we utilized Joule heating to achieve precise control over the formation of metal oxide phases and their distribution across metallic patterns. For this study, we used thick Mo wires with a thickness of 400–450 nm. While we tested a few samples with thinner metal, we found that Joule heating could not consistently or reproducibly oxidize these thinner wires, as they would quickly burn out, resulting in an open circuit. Our findings, using the Photoluminescence Thermometry method, reveal that the temperature of the wires ranged from 230 °C to 300 °C, depending on the applied power. These temperatures were adequate to induce oxide formation on molybdenum (Mo) wires, leading to extensive areas of molybdenum oxides on and around the Mo-sputtered wires. This demonstrates the potential of Joule heating as an effective tool for spatially controlled oxide growth, paving the way for subsequent thin-film TMD development. To gain detailed insight into the specific oxides present in the samples, Raman spectroscopy was utilized to characterize the samples at each stage. Distinct peaks related to different types of molybdenum oxides appeared under the respective conditions, providing valuable insights into the oxidation process. The analysis of oxide formation during Joule heating revealed that the applied power and heating duration significantly influence the resulting oxide phase. For example, at powers lower than 2 W, even over long durations, no oxides were detected, indicating that these conditions are insufficient for measurable oxide formation. However, at powers of 2 W and above, as shown in Figure 2A, oxide formation occurs in significant quantities within a 2 s duration. The Raman spectra in Figure 2A(a1–a3) confirm that MoO_2_ is the primary product at powers of 2, 2.5, and 3 W, respectively. Raman peaks at 354, 457, 493, 568, and 738 cm^−1^ are observed, which correspond well with reported values in the literature [21,29,30]. As the power increases, the optical images and Raman spectra reveal an increase in the amount of oxide formed and a variation in oxide types, transitioning from MoO_2_ to a mixture of oxides, as shown in Figure 2A(a4,a5). In (a4), MoO_3_ peaks emerge at 819 and 994 cm^−1^ [24] alongside MoO_2_ peaks, while (a5) also indicates the presence of Mo_4_O_11_, with Raman peaks observed at 450, 791, 904, and 940 cm^−1^ [21,22,23,24,25,26,27,28,29,30]. This suggests that higher power levels not only promote more extensive oxidation but also contribute to the formation of increasingly complex oxide phases, simultaneously expanding the spatial range of oxide growth.

**Figure 3:**
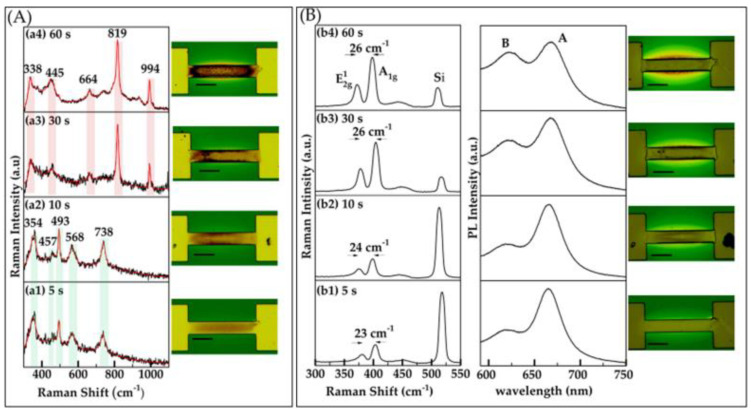
(**A**) Raman characterization and optical images of oxide layers formed on Mo patterns induced by Joule heating. Oxide formation was investigated as a function of duration, with a fixed duration of 2 s. Raman spectra were recorded with a power of 1.5 mW and an integration time of 15 s. This section includes four sub-panels (**a1**–**a4**) displaying Raman spectra corresponding to different oxide formations from varying applied power levels, along with optical images of the oxide regions. (**B**) Raman, PL spectra, and optical images of the sample after sulfurization. This section includes four sub-panels (**b1**–**b4**), each displaying Raman and PL spectra along with corresponding optical images that illustrate the structural and spectral evolution of MoS_2_. Each optical image features a scale bar representing 50 μm.

To examine the influence of heating duration on oxide formation, we investigated the impact of varying heating times. Specifically, we maintained a constant power of 2.5 W while adjusting the duration of heating to 5, 10, 30, and 60 s. Our results indicate that for shorter durations, such as 5 and 10 s, MoO_2_ was predominantly formed and localized within the desired regions. Raman peaks at 354, 457, 493, 568, and 738 cm^−1^ were observed [21,29,30], as shown in Figure 3A(a1,a2). However, as the heating time increased to 30 s and 1 min, as shown in Figure 3A(a3,a4), respectively, MoO_3_ started to emerge with associated Raman peaks at 664, 819, and 994 cm^−1^ [24]. This suggests that longer heating times promote further oxidation, leading to the transition from MoO_2_ to MoO_3_.

The sulfurization of oxidized regions resulted in the growth of MoS_2_, driven by the reaction of sulfur vapors with MoOₓ formed during the oxidation process. This reaction initiates at the oxidized regions and propagates outward along the substrate, as illustrated in the optical images in Figure 2B and Figure 3B. This growth mechanism closely resembles a chemical vapor deposition (CVD) model described in our previous studies [20,21], where a thin metal oxide layer on the metallic patterns serves as a precursor. The chalcogen reacts with the metal oxide, leading to the formation of highly crystalline MoS_2_ films that diffuse outward along the substrate while remaining anchored to the lithographically defined contacts. Furthermore, the amount of material growth was observed to increase with the applied power and the duration of the oxidation process. Higher power and longer oxidation times resulted in thicker MoS_2_ layers, particularly around the metal contacts, highlighting the critical role of these parameters in controlling the spatial distribution and thickness of the MoS_2_ material. As the material extends further from the metal, it gradually transitions to thinner layers.

We performed optical characterizations of the grown MoS_2_ using Raman and photoluminescence (PL) spectroscopy to evaluate the quality of the material, as shown in Figure 2B and Figure 3B. The Raman spectra display two characteristic peaks corresponding to the $E_{2g}^{1}$ and $A_{1g}$ vibrational modes, representing the in-plane and out-of-plane vibrations, respectively. Importantly, the separation between these peaks serves as an indicator of the material’s thickness [23]. The PL spectra show two excitonic emission peaks, A and B, showcasing the material’s excellent optical properties and structural quality.

Building on our optical characterization, low power (2–3 W) oxidation results in few-layer MoS_2_ growth, indicated by the separation between the $E_{2g}^{1}$ and $A_{1g}$ vibrational modes (~22–24 cm^−1^), as shown in Figure 2B(b1–b3). As the power increases (3.5 W and above), a comparatively higher Raman shift difference is observed (≥25 cm^−1^), as shown in Figure 2B(b4,b5). Similarly, for duration times of 5–10 s, few-layer MoS_2_ is observed, with a separation of (~23–24 cm^−1^) between Raman vibrational modes, as shown in Figure 3B(b1,b2). As the oxidation time extends to 30–60 s, a bulk-like structure is evident, with a Raman shift separation of 26 cm^−1^, as shown in Figure 3B(b3,b4). The PL measurements align with the Raman findings, revealing a signal characteristic of few-layer-like structures at lower power and shorter durations, transitioning to bulk-like structures as the power and duration increase, as depicted in Figure 2B and Figure 3B. Additionally, SEM images of some oxidized and corresponding sulfurized areas, obtained using our filament-based Tescan VEGAII, reveal a surface topology consistent with oxide formation in the channel. These images and detailed descriptions are provided in the Supplementary Materials to further support our findings [31,32].

**Figure 4:**
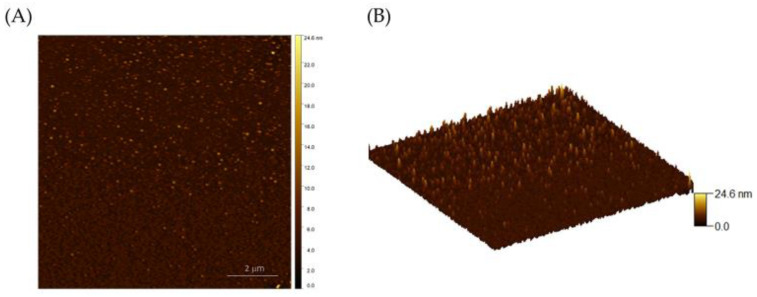

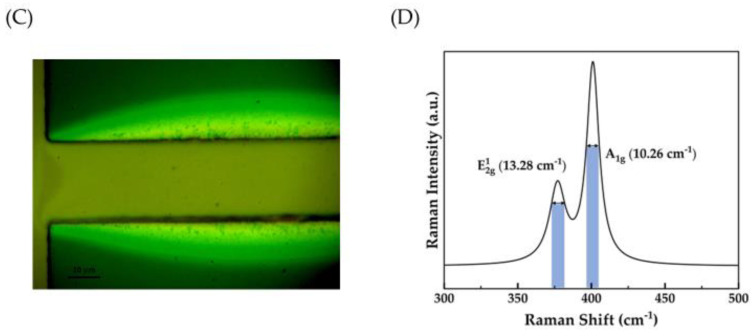
(**A**,**B**) Two-dimensional and three-dimensional AFM images of grown MoS_2_ film, respectively. (**C**) An optical image of MoS_2_ film. (**D**) Raman spectra of MoS_2_ illustrating the FWHM.

Atomic force microscopy was used to analyze the surface morphology and continuity of MoS_2_ films. Figure 4A,B show 2D and 3D AFM images of the MoS_2_ film, respectively. Figure 4C shows an optical image of a MoS_2_ film produced by sulfurizing the oxide at 3 W for 2 s. The crystallinity of the MoS_2_ films was evaluated by the full width at half maximum (FWHM) of the Raman spectra. As displayed in Figure 4D, Raman peaks were found at 377 cm^−1^ and 400 cm^−1^, which correspond to the $E_{2g}^{1}$ in-plane and $A_{1g}$ out-of-plane vibrational modes of MoS_2_. The observed FWHMs for the $E_{2g}^{1}$ and $A_{1g}$ peaks were 13.28 cm^−1^ and 10.26 cm^−1^, suggesting good crystallinity according to the literature [33].

## 4. Conclusions

This study demonstrates the efficiency of using Joule heating in the controlled oxidation of molybdenum metallic patterns and subsequent transformation into MoS**_2_** through the sulfurization process. Raman spectroscopy indicated that we successfully controlled the formation of different types of molybdenum oxides, primarily MoO_2_ and MoO_3_, by varying the power and duration time. The oxidation process was found to be highly spatially localized, with the type and thickness of the oxide directly influenced by the applied power and heating duration. This control allowed the precise spatial growth of MoS_2_, with a thickness gradient ranging from bulk-like structures near the oxidized regions to few-layer formations farther away. Significant differences in Raman spectral shifts, particularly with increasing power and oxidation time, indicated a transition from few-layer material to bulk structures. These findings were further supported by photoluminescence measurements. The results indicate that the Joule heating technique can be a promising method for localized material manipulation in two-dimensional transition metal dichalcogenides. Future studies should focus on optimizing heating parameters and incorporating an inert atmosphere to reduce defect density and improve the crystalline quality of synthesized MoS**_2_** films.

## Data Availability

The raw data supporting the conclusions of this article will be made available by the authors on request.

## Supplementary Materials

The following supporting information can be downloaded at https://www.mdpi.com/article/10.3390/nano15020131/s1: Figure S1: Photoluminescence spectrum of Er_2_O_3_ at temperature of 25 °C (black) and at 450 °C (red). Figure S2: The natural logarithm of the relative peak areas from the H (539 nm) and S (564 nm) bands as a function of inverse temperature. Figure S3: The calculated temperature of Mo wire as a function of applied power. Figure S4: SEM images of the oxidized (left) and corresponding sulfurized (right) areas obtained using a Tescan VEGAII system. Panels (a), (b), and (c) show results for a duration of 2 s at applied powers of 2.5 W, 3 W, and 3.5 W, respectively. Panels (d) and (e) display results for an applied power of 2.5 W with durations of 10 s and 30 s, respectively. The scale bar representing 1 μm. Figure S5: Field Emission Scanning Electron Microscopy (FESEM) images of the sample, taken using a Hitachi S-4500, illustrating the lateral growth of 2D MoS_2_ from lithographically defined molybdenum metal patterns toward the Si/SiO_2_ substrate. The central image provides an overview of the patterned metal, while the magnified images highlight the flake-like morphology of the grown MoS_2_ material, with laminate-like features extending over areas up to 100 µm in size.
