# Supplementary material for: Controlled Oxidation of Metallic Molybdenum Patterns via Joule Heating for Localized MoS2 Growth
Source: Nanomaterials (Basel). 2025 Jan 16;15(2):131. doi: 10.3390/nano15020131 (PMC11767446; doi:10.3390/nano15020131)
Supplement: Supplementary file 1 [file nanomaterials-15-00131-s001.zip › nanomaterials-3408121-supplementary.pdf]

nanomaterials

Supplementary Information

Controlled Oxidation of Metallic Molybdenum Patterns via Joule Heating for Localized MoS_2 Growth

Norah Aldosari [1,2,3], William Poston [1,2], Gregory Jensen [1,2], Maryam Bizhani [1,2], Muhammad Tariq [1,2] and Eric Stinaff [1,2,*]

[1] Department of Physics and Astronomy Athens, Ohio University, Athens, OH 45701, USA; na314617@ohio.edu (N.A.); wp826418@ohio.edu (W.P.); gj772812@ohio.edu (G.J.); mb795116@ohio.edu (M.B.); mt967721@ohio.edu (M.T.)
[2] Nanoscale and Quantum Phenomena Institute (NQPI), Athens, OH 45701, USA
[3] Department of Physics and Astronomy, College of Science and Humanities, Prince Sattam bin Abdulaziz University, 173, Al-Kharj 16278, Saudi Arabia
* Correspondence: stinaff@ohio.edu

Academic Editor(s): Christian Mitterer

Received: 18 December 2024
Revised: 9 January 2025
Accepted: 14 January 2025
Published: date

Citation: Aldosari, N.; Poston, W.; Jensen, G.; Bizhani, M.; Tariq, M.; Stinaff, E. Controlled Oxidation of Metallic Molybdenum Patterns via Joule Heating for Localized MoS_2 Growth. *Nanomaterials* **2025**, *15*, x. https://doi.org/10.3390/xxxxx

Copyright: © 2025 by the authors. Submitted for possible open access publication under the terms and conditions of the Creative Commons Attribution (CC BY) license (https://creativecommons.org/licenses/by/4.0/).

Temperature Estimation Using Er_2O_3 as Temperature Probes

To estimate the temperature during Joule heating experiments, we utilized Er_2O_3 as local temperature probes. This method is based on the temperature-dependent photoluminescence (PL) properties of Er_2O_3, which enables absolute local temperature measurement via changes in the populations of thermally coupled energy levels ($^2H_{11}/_2$ and $^4S_3/_2$) of the Er^{3+} ion with change the temperature [27,28]. Figure S1showes photoluminescence spectrum of Er_2O_3 at different temperatures.

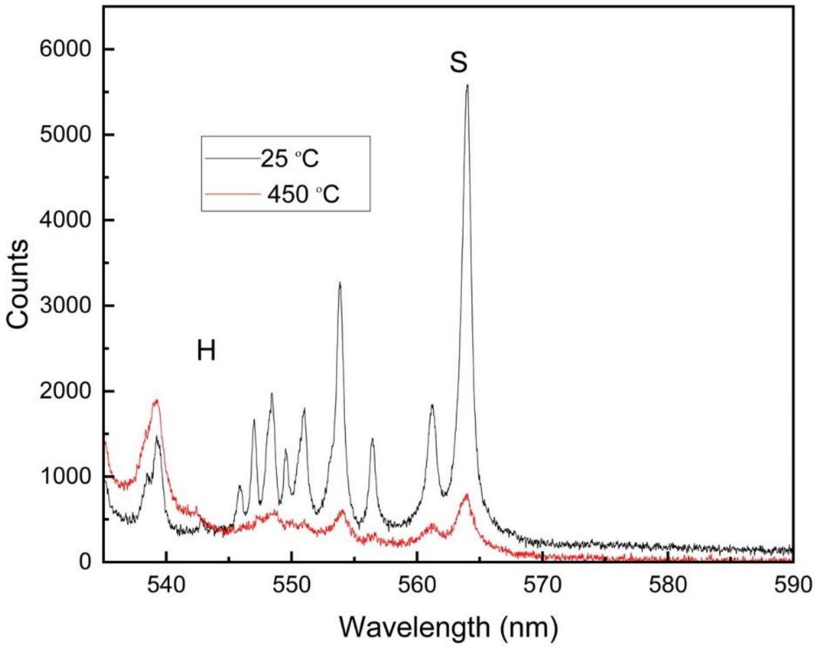

Figure S1. Photoluminescence spectrum of Er_2O_3 at temperature of 25C (black) and at 450 C (red).

Preparation of Er_2O_3 Solution and Deposition

Er_2O_3 powder was first dissolved in toluene, followed by sonication to achieve a uniform suspension. This solution was then drop-cast onto lithographically prepared molybdenum (Mo) wires on Si/SiO_2, which were subsequently left to dry. The prepared Mo wires with Er_2O_3 were connected to a current source and placed under a 4x microscope objective for PL measurements. Initial photoluminescence spectra of the Er_2O_3 on the Mo wire were recorded at room temperature. Current was then applied through the Mo wire, causing Joule heating, and the PL spectra were measured at different intervals to capture temperature-dependent changes. To accurately calibrate the temperature response, the same Er_2O_3 solution was drop-cast onto a Si/SiO_2 substrate. This substrate was affixed to a ceramic heater, and a type k thermocouple thermometer was attached to the substrate's surface to provide precise temperature measurements. The temperature of the ceramic heater was gradually increased, and the PL spectra of the Er_2O_3 nanoparticles were recorded at known temperatures. The photoluminescence spectra are shown in Figure S1. The laser excites the Er_2O_3 particle, resulting in an emission from the transition at 539 nm (labeled as H) and 564 nm (labeled as S). The relative population of various states is represented by the peak areas of the H and S transitions. Since temperature influences state populations, the absolute temperature can be determined by measuring and analyzing the relative emission intensities, applying Boltzmann statistics $\frac{H}{S} = A \exp\left(-\frac{\Delta E}{KT}\right)$ [27,28]. A plot of the natural logarithm of H/S versus the reciprocal temperature produces a straight line, as in figure S2, with the slope representing the value of $-\frac{\Delta E}{K}$ and the intercept corresponding to the natural logarithm of A [27,28].

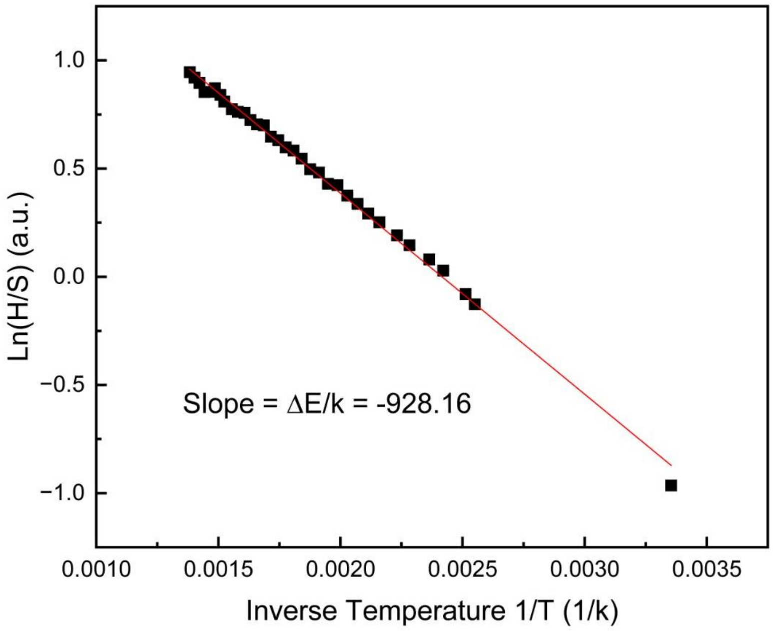

Figure S2. The natural logarithm of the relative peak areas from the H (539 nm) and S (564 nm) bands as a function of inverse temperature.

The slope is equal to -928.12 ± 10.33 with an intercept equal to 2.24 ± 0.02. Hence, the Boltzmann expression is $\frac{H}{S} = 9.39 \exp\left(\frac{-928.16}{T}\right)$, and an absolute temperature is $T = \frac{928.16}{2.24 - \ln\left(\frac{H}{S}\right)}$. This calibration was essential for interpreting the temperature changes during the Joule heating of the Mo wires. We employed this technique to calculate the temperature of the Mo wire under varying applied power levels. The results, depicted in Figure S3, illustrate the relationship between temperature and power.

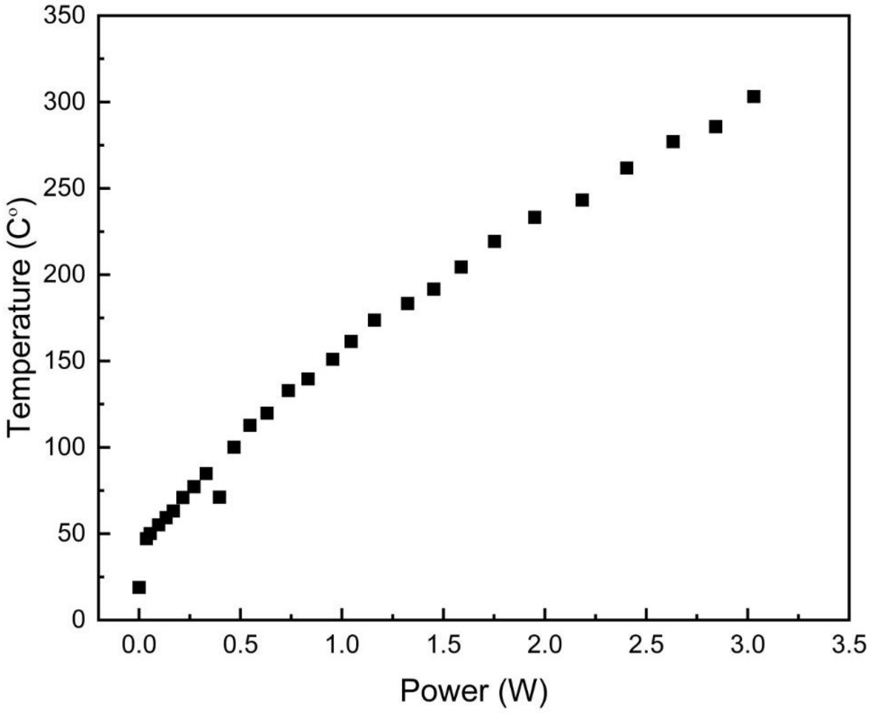

Figure S3. The calculated Temperature of Mo wire as function of applied power.

SEM and FESEM Analysis of Oxidized and Corresponding Sulfurized Areas:

Scanning Electron Microscopy (SEM) was utilized to investigate the surface topology of both the oxidized and corresponding sulfurized regions. The SEM images were acquired using a filament-based Tescan VEGAII system.

The SEM images of the oxidized areas exhibit a distinct surface texture, characterized by a granular structure indicative of molybdenum oxide formation as found in the literature [32,33]. These features suggest uniform oxidation of the metallic regions, with the topology varying slightly depending on the applied power and oxidation duration. Higher power and longer oxidation times result in the formation of a thicker oxide layer, as evident from the increased surface roughness and more pronounced granular features in the SEM images. Corresponding Sulfurized Areas: Following sulfurization, the SEM images reveal a change in surface morphology. The granular features observed in the oxidized areas transition into smoother or layered structures, consistent with the formation of MoS_2.

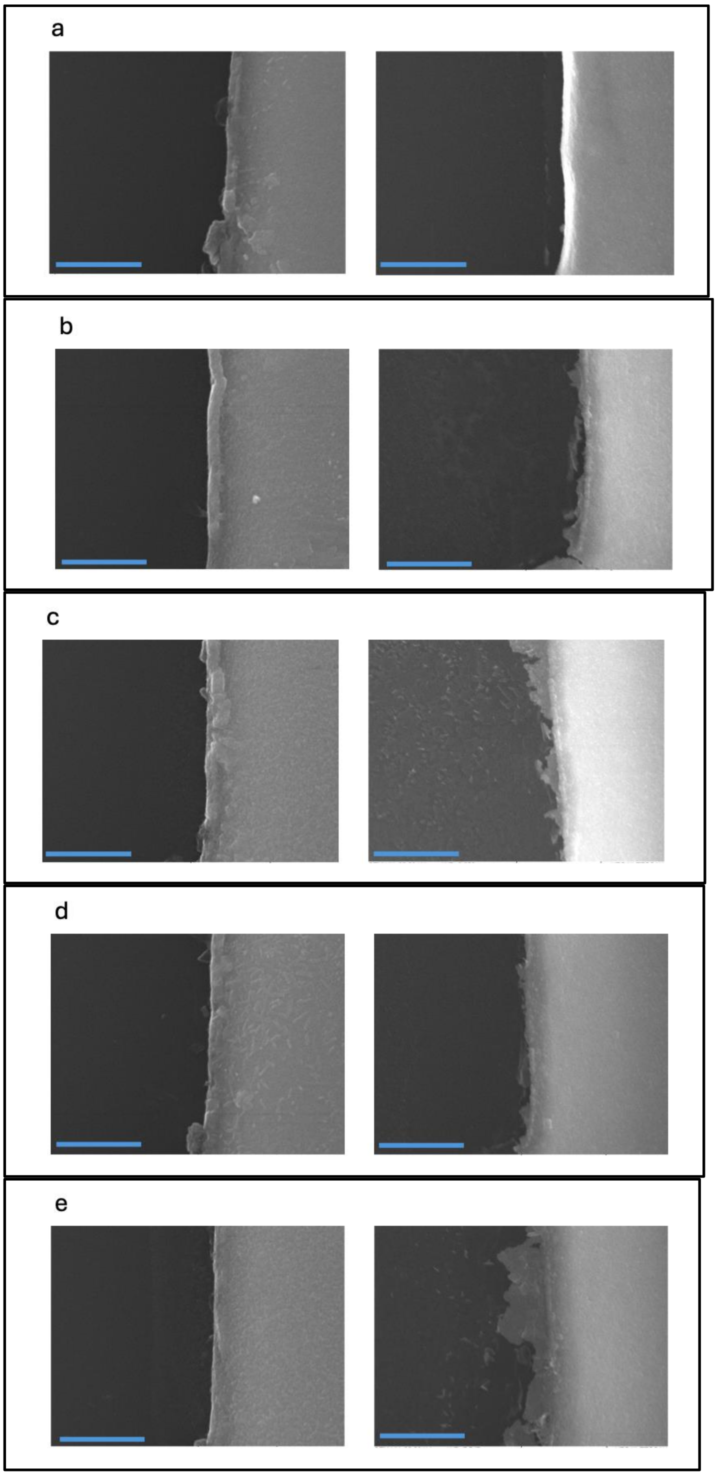

Figure S4. SEM images of the oxidized (left) and corresponding sulfurized (right) areas obtained using a Tescan VEGAII system. Panels (a), (b), and (c) show results for a duration of 2 seconds at applied powers of 2.5 W, 3 W, and 3.5 W, respectively. Panels (d) and (e) display results for an applied power of 2.5 W with durations of 10 seconds and 30 seconds, respectively. The scale bar representing 1 μm.

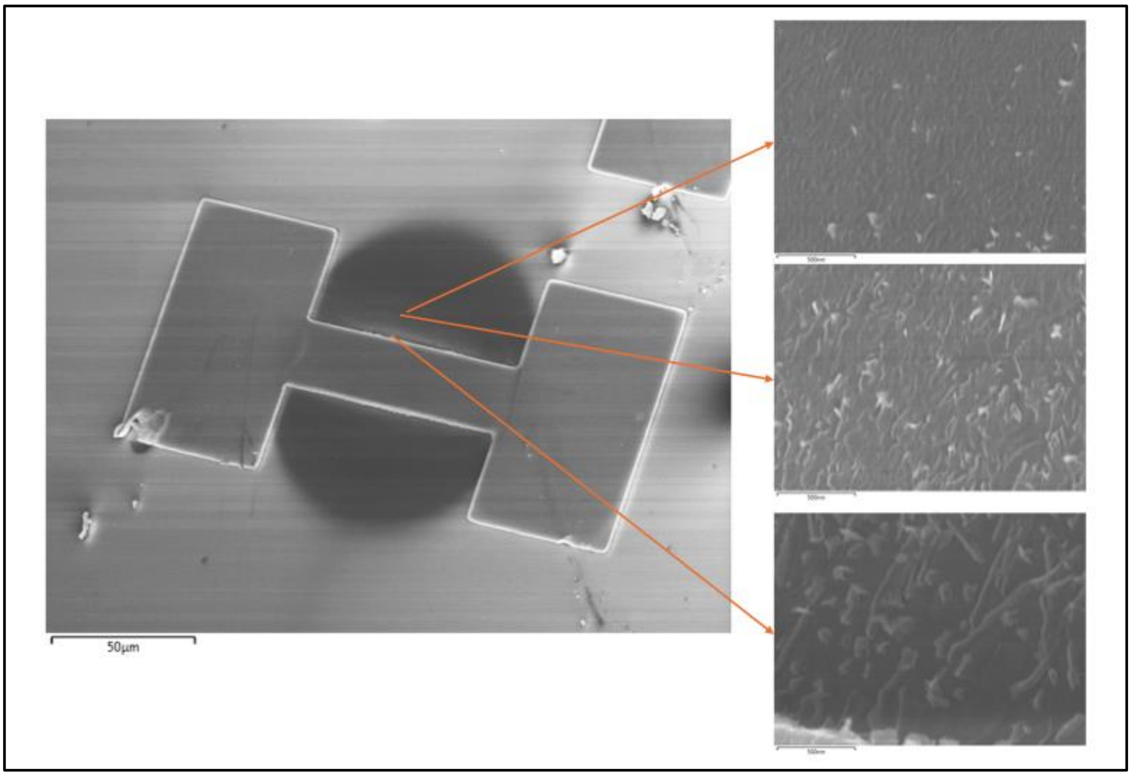

Figure S5. Field Emission Scanning Electron Microscopy (FESEM) images of the sample, taken using a Hitachi S-4500, illustrating the lateral growth of 2D MoS_2 from lithographically defined molybdenum metal patterns toward the Si/SiO_2 substrate. The central image provides an overview of the patterned metal, while the magnified images highlight the flake-like morphology of the grown MoS_2 material.
